# Chemistry Laboratories Make Tons of Plastic Waste. Can We Recycle It?

**DOI:** 10.1021/acscentsci.6c00139

**Published:** 2026-01-30

**Authors:** Victoria Atkinson

## Abstract

Chemists and innovative recyclers are trying to put gloves, pipet tips, and other laboratory plastic waste to use.

## Graphical abstract

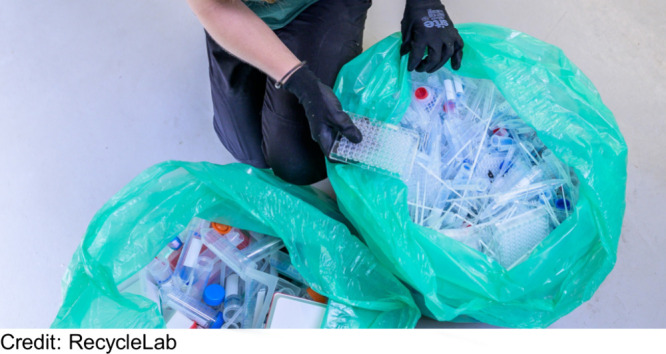

It’s almost impossible to imagine working in a modern lab without plastic. We instinctively reach for disposable gloves, pipet tips, and plastic syringes. These single-use consumables minimize the risk of contamination and save tedious hours of washing up.

“Single-use plastics are one of those necessary evils,” says Kristen Weeks, who recently received her PhD from Florida State University (FSU). “We do science in order to justify a lot of different political decisions about the environment and how we function as a society, but there’s irony in the fact that we have to use disposable plastics to do it.”

The majority of plastic lab items are not accepted for recycling because of their complexity and potential contact with harmful chemicals; instead, they undergo incineration or pyrolysis. But for Weeks and many other young scientists, the belief that we could and should do better is inspiring grassroots recycling projects worldwide. They’re partnering with innovative plastic processors to tackle chemistry’s plastic waste problem.

## The rippling impact of student initiatives

Entering the lab for the first time in 2014, College of Charleston undergraduates Evan Bailey and Caroline Gilmer were astonished by the sheer volume of disposable plastic involved in practical work, particularly gloves. With more than 1,000 students enrolled in lab classes, they figured that the chemistry and biochemistry departments alone were going through a staggering 54,000 gloves each semester. That’s more than 180 kg of plastic waste.

In 2017, Bailey and Gilmer got to work drafting a project proposal and applying for a $5,000 grant from the college’s environmental education-focused ECOllective Student Projects Committee. Bailey and Gilmer’s recycling program launched in 2018. After researching different options, they chose the private waste management company TerraCycle. They bought and distributed 36 of the firm’s recycling boxes around the science buildings.

**Figure:**
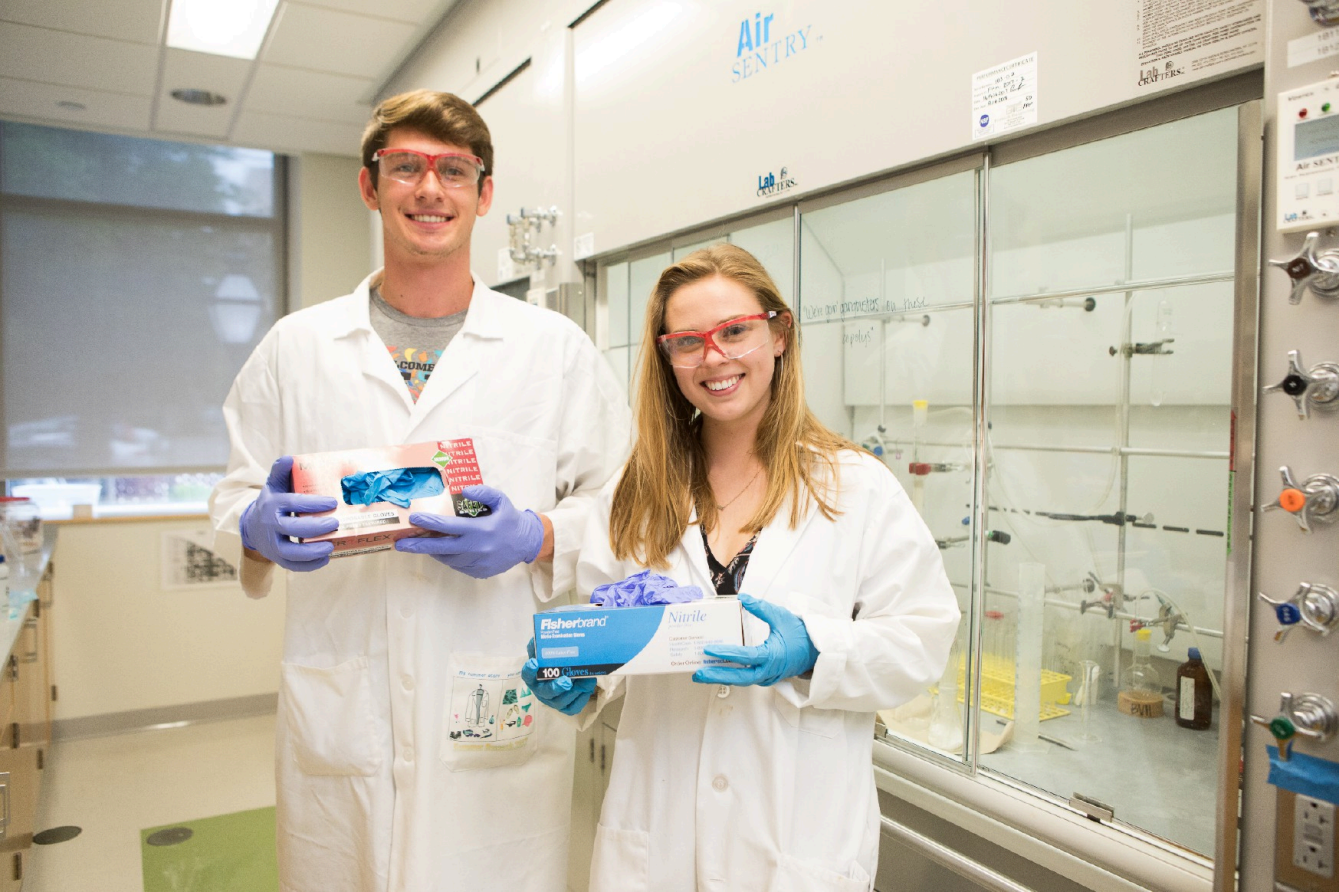
Evan Bailey and Caroline Gilmer were inspired to begin the glove recycling initiative at College of Charleston when they realized how much plastic is generated by laboratory science. Credit: Reese Moore/College of Charleston.

On the university’s end, TerraCycle’s process is straightforward: the university orders special recycling boxes from the company website as needed and ships the boxes back when they’re full. TerraCycle uses its recycling systems to turn the used plastic into a variety of new products. But the hefty up-front cost of a box—$311 for a medium, which includes processing and shipping—can be a barrier to initiating such a plan.

The biggest challenge is ensuring that the effort is sustainable, says Kate Mullaugh, an analytical environmental chemist at the college and the recycling program’s faculty sponsor. Over the 7 years that it has been operating, “making sure that we have the program funded has been a struggle,” Mullaugh says. “We’re taking on an extra cost to recycle these gloves, but like a lot of other chemistry departments, we’re struggling with the ever-increasing costs of lab operation.”

Though the cost is high, so is the impact. The program inspires and empowers a new generation of students, Mullaugh says. Since Bailey and Gilmer graduated, she has been the permanent point of contact for the program—though its day-to-day management remains in student hands—and she is never short of volunteers.

“Labs are not the first place you think of for recycling materials, so I think it opened people’s minds up to the idea that you could recycle at least some of the plastic waste that was being generated. The students are really excited to participate in this,” Mullaugh says. “I’ve been encouraged to hear from people like Kristen [Weeks] that have gone elsewhere and taken this idea with them—there are ripple effects from these initiatives.”

As an undergraduate, Weeks volunteered with the Charleston program for a couple of years before starting graduate school at FSU in 2020. The next summer, she and fellow graduate student Carley Reid conducted an audit on single-use plastic consumption within the chemistry department. The results were pretty dire: their survey revealed a monthly average of almost 16,000 pairs of gloves, 6,500 pipet tips, and 4,000 syringes.

“While we were discussing those results, I brought up the fact that I was familiar with a program that could mitigate this problem,” Weeks says. “We realized the data that we had gathered was going to make a really excellent case for implementing a glove recycling project.” The two quickly drafted a project proposal and, with the support of several department professors, applied for funding through FSU’s sustainable campus office.

Within a year, the Student Sustainability Team had deployed TerraCycle boxes across each floor of the chemistry research buildings, with volunteers monitoring and consolidating boxes on a monthly basis. “Initially, a lot of people had questions: What’s allowed? Is it safe to mix plastics with different chemicals on them?” Weeks says. “We had a lot of signage.”

**Figure:**
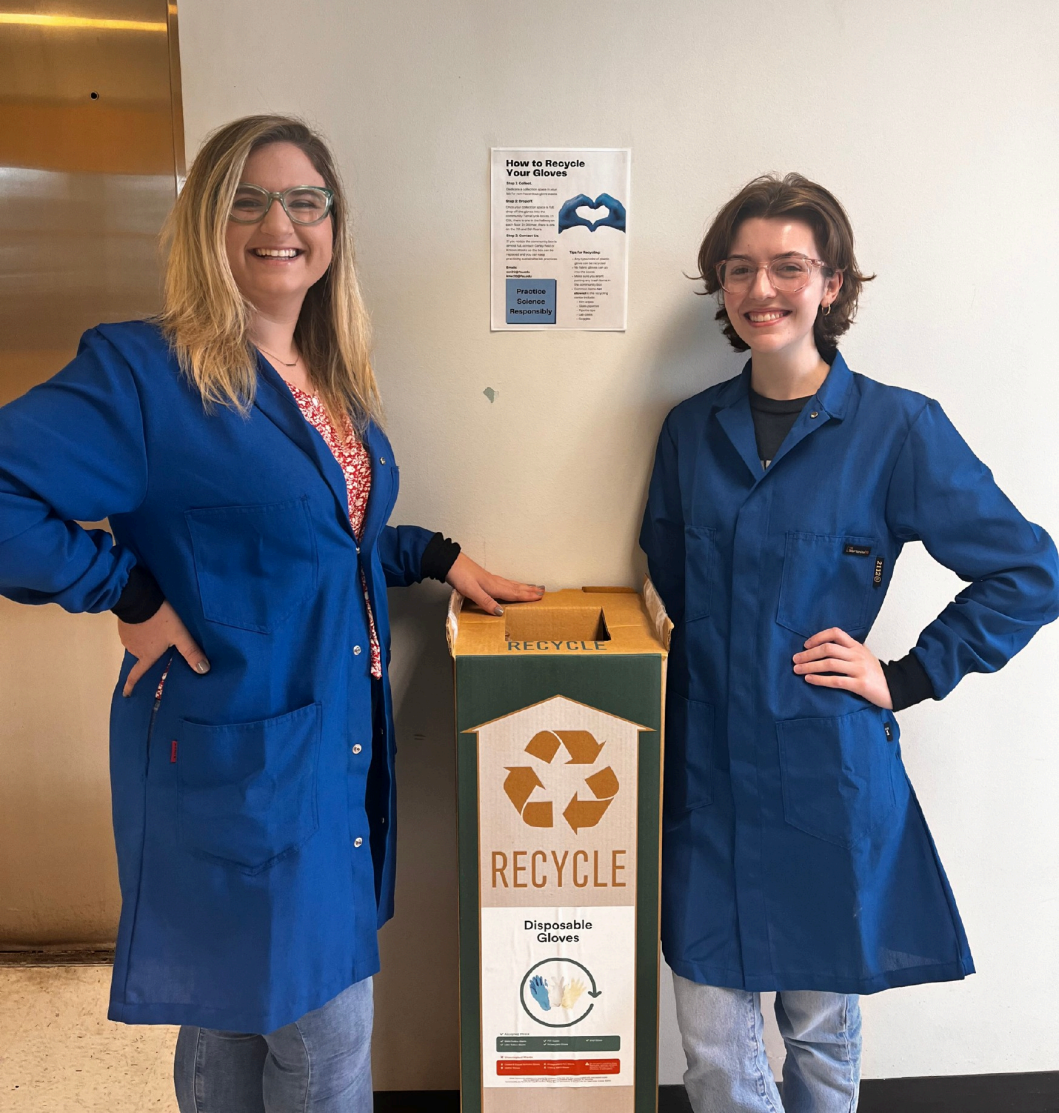
Kristen Weeks (left) and Carley Reid established a lab waste recycling program in the graduate chemistry laboratories at Florida State University. Credit: Florida State University.

The team targeted lightly used lab gloves and delineated what could and could not be put in the boxes. “If you’re using specific chemicals, like biosafety level 2”—biological agents that pose a moderate disease hazard but aren’t transmitted through airborne exposure—“they are not allowed, and we didn’t implement glove recycling in those labs,” Weeks says.

After the team addressed fellow department members’ concerns about the operation and its safety, the initiative received an enthusiastic response. Almost 90 kg of gloves were recycled in the first year.

This success attracted the interest of other science departments on campus. Weeks and Reid helped them implement similar programs in 2023 and took steps to secure the initiative’s long-term future in the chemistry department. They passed the program’s management to university staff before they graduated.

## How to grind up gloves

These initiatives are a relatively recent addition to many universities, but TerraCycle has been developing and improving methods to process lab gloves since the early 2010s. The New Jersey-based company, which now operates in more than a dozen countries, was founded in 2001 with the goal of developing processes to recycle the unrecyclable, according to TerraCycle head scientist Ernel Simpson. “I’ve worked on cigarette filters, diapers, chewing gum,” he says.

“The way TerraCycle is arranged as a company, we don’t own production equipment; we do R&D,” Simpson says. The firm approaches processors that have the equipment it’s looking for and then develops a recycling process to meet the needs of the client and the market. The partner model lets TerraCycle start up a process more quickly and at scale.

“Twelve years ago, a number of rubber glove manufacturers came to us. They had piles and piles of gloves—not only used, but prototypes and blanks,” Simpson says. His team had to create a method to recycle the mountain of gloves. But to do that, it would have to overcome the innate properties of nitrile rubber.

Nitrile rubber—a copolymer of acrylonitrile and butadiene—is extremely tolerant to a wide range of temperatures and a variety of chemicals. The extensive cross-linking between the polymer chains gives the rubber the strength and flexibility that make it ideal for laboratory gloves.

These properties have made the material a valuable lab staple. But they also make nitrile challenging to convert into a powder form—a requirement for refabrication into new products.

After a year of research, TerraCycle deployed its first process. First, an optical sorter separated nitrile gloves from any latex or polyvinyl chloride gloves in the waste stream. They then froze the sorted gloves to cryogenic temperatures, then ground the now-brittle items into fine particulates. The resulting rubber powder was melted and reformulated with appropriate additives to create high-quality recycled products, including park furniture, roof tiles, and nitrile rubber sheeting.

TerraCycle has seen a dramatic increase in demand and now handles an estimated 2.6 million gloves annually across 22 countries. The process itself is constantly under development, Simpson says.

With the increase in demand, TerraCycle has upgraded the micronization process, switching the freeze-grind sequence for newer jet milling technology. High-speed jets of compressed gas blast the plastic particles into one another, breaking them down with each collision to create an ultrafine powder. The process works well for the hard-to-grind rubber and uses less energy than the previous grinding approach.

“The objective is to develop processes that are sophisticated enough to get good products at a cost that is viable for everyone,” Simpson says.

## Tackling waste beyond gloves

Although gloves are the most abundant type of plastic waste generated in the lab, they are just one of many. Mixed contaminated materials such as pipet tips and syringes present more of a challenge to industrial handlers like TerraCycle.

It’s with such materials that smaller specialist companies like UK-based RecycleLab find their niche. “There’s a perceived risk within a science lab that waste management companies won’t touch. Syringes and tips particularly are classed as hazardous when in reality, many are not necessarily,” says RecycleLab CEO Danielle Stephens. “These items are small too, so if they do get taken to a general waste management facility, they just slip through the machinery.”

**Figure:**
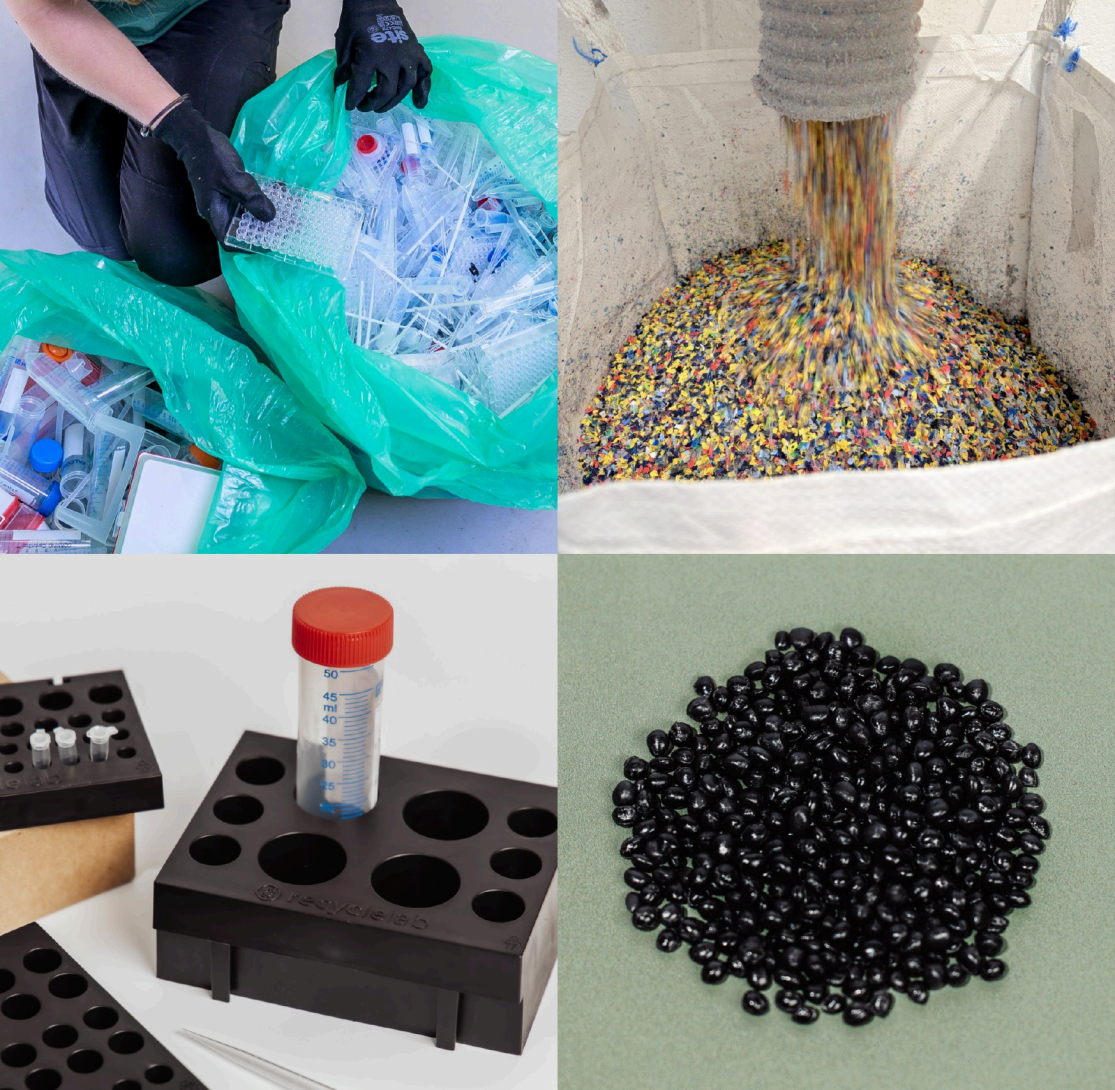
Clockwise from top left, RecycleLab sorts used plastic products by hand, shreds the plastics, turns them into pellets, and melts them down into new products such as sample holders. Credit: RecycleLab.

A biochemist herself, Stephens witnessed firsthand the scale of science’s plastic problem and founded RecycleLab in 2021. The company offers a bespoke service, performing a lab audit to evaluate how recycling procedures could fit into an individual group’s workflow. That includes providing a thorough report on current waste volumes, their associated carbon emissions, and alternative greener disposal options.

“Generally after an audit, the labs will look at how they can increase the diversion rate of the materials away from incineration and to alternative waste streams such as recycling,” Stephens says. This can involve simple strategies such as placing RecycleLab’s bins in the lab, but the company also works with the site health and safety teams to educate scientists on whether certain waste is actually hazardous or can be placed into a conventional recycling stream.

Filled cardboard bins are shipped back to RecycleLab, which is unusual in accepting decontaminated as well as uncontaminated plastic. “Most of the time there’s no change in protocols whatsoever—if they’re using a biological hazard or working with [genetically modified organisms], for example, they’re already decontaminating before the waste goes off-site,” Stephens says. “Instead of putting it into general waste or back into clinical waste, it can go into our recycling bin.”

RecycleLab manually sorts this waste by polymer type—principally polypropylene, polystyrene, polyethylene terephthalate, and high-density polyethylene—and shreds the separated material using a granulator. The resulting flakes are extruded into pellets, which are then sold to manufacturers to make into new products.

RecycleLab is working with a company that makes recyclable bathroom and kitchen accessories. “And we manufacture test-tube racks ourselves so that we can sell this back to the science industry,” Stephens says. “We’re also testing our material with equipment suppliers to get it back into lab consumables.”

This transparent and tailored approach attracted the University of Oxford’s Sir William Dunn School of Pathology to RecycleLab. “Staff are motivated when they see their waste becoming something tangible like a tip box or tube rack,” says Saroj Saurya, a postdoctoral researcher and head of the Dunn School’s sustainability-focused Green Group. “It was important to us to know that the plastics really were being recycled into useful products, not just exported or downcycled.”

**Figure:**
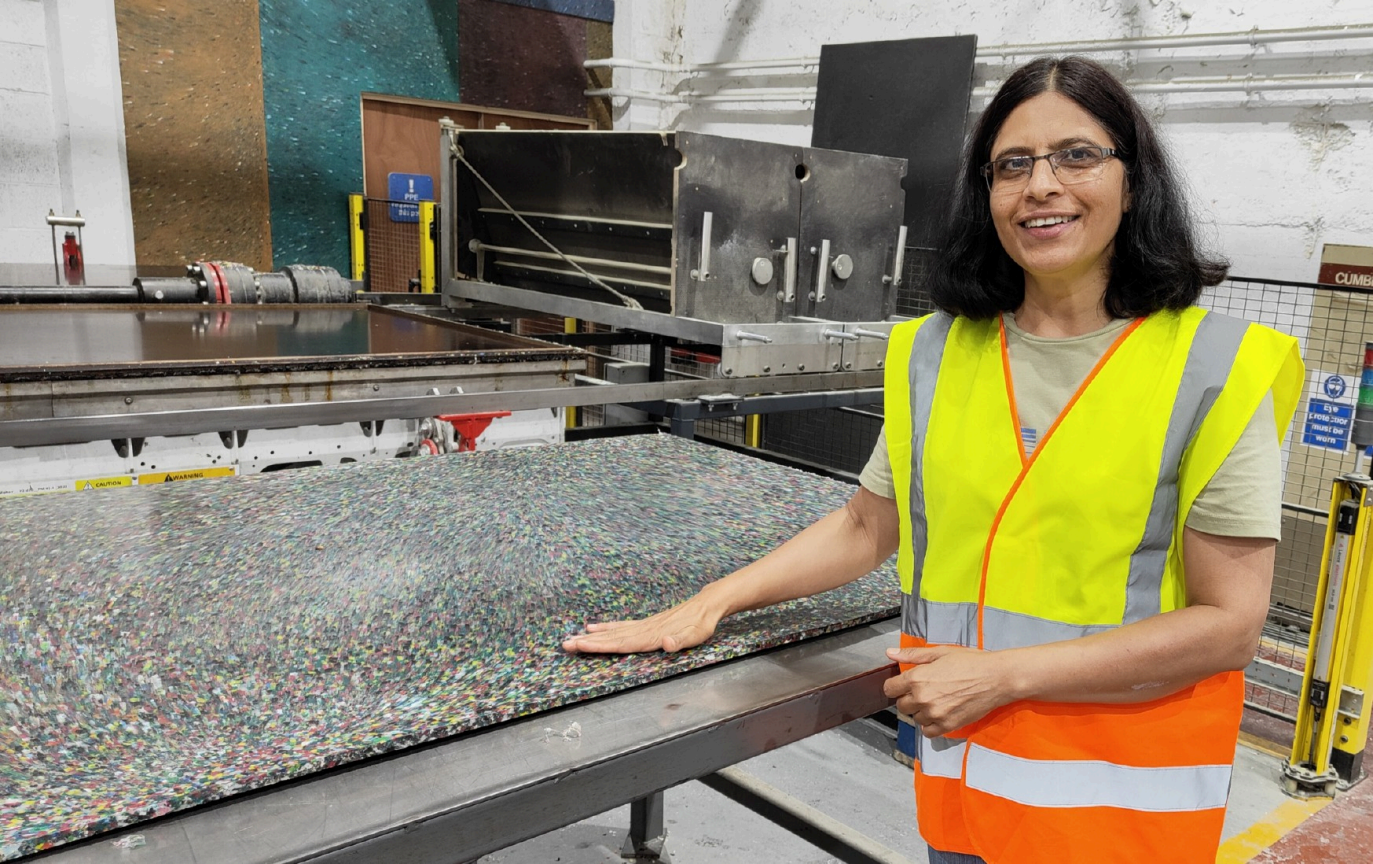
Saroj Saurya, above, and her team at the University of Oxford recycle lab products through several waste management companies. MYGroup’s facilities shred, grind, and melt used lab plastic to be made into new products. The recycled plastic ply shown here can be used as a building material. Credit: Luke Housley.

Similarly concerned by the volume of plastic consumables going to incineration, the Green Group piloted a glove recycling program with a generic recycler in 2020. The group expanded its efforts with RecycleLab—first to uncontaminated plastics, then to chemically decontaminated plastics, and finally to autoclaved plastics in 2024.

“Department-wide, we now recycle around 500 kg of decontaminated lab plastics and 400 kg of uncontaminated gloves annually,” Saurya says. “In the last 5 years, we have recycled close to a [metric] ton of plastic that would otherwise have been incinerated.”

The technology and processes needed to recycle a number of lab consumables already exist; for many scientists, the challenge remains administrative rather than technical. But the commitment of the latest generation of chemists to more sustainable science is already driving change.

“Younger researchers especially want their work to align with their values. Over time, this became a departmental culture shift,” Saurya says. “We’ve shown that with volunteers, clear systems, and the right partners, much more can be recycled safely.”

*Victoria Atkinson is a freelance contributor to* Chemical & Engineering News, *the independent news outlet of the American Chemical Society.*

